# Preservation of human heart valves for replacement in children with heart valve disease: past, present and future

**DOI:** 10.1007/s10561-023-10076-2

**Published:** 2023-02-01

**Authors:** M. C. Peters, B. P. T. Kruithof, C. V. C. Bouten, I. K. Voets, A. van den Bogaerdt, M. J. Goumans, A. van Wijk

**Affiliations:** 1 Department of Pediatric Cardiothoracic Surgery, Wilhelmina Children’s Hospital, University Medical Center Utrecht, 3584 EA Utrecht, The Netherlands; 2 Department of Cardiovascular Cell Biology, Leiden University Medical Center, 2300 RC Leiden, The Netherlands; 3 Department of Cardiology, Leiden University Medical Center, 2333 ZA Leiden, The Netherlands; 4 Department of Biomedical Engineering, Eindhoven University of Technology, 5600 MB Eindhoven, The Netherlands; 5 Institute for Complex Molecular Systems, Eindhoven University of Technology, 5600 MB Eindhoven, The Netherlands; 6 Department of Chemical Engineering and Chemistry, Eindhoven University of Technology, 5600 MB Eindhoven, The Netherlands; 7 Heart Valve Department, ETB-BISLIFE Multi Tissue Center, 2333 BD Beverwijk, The Netherlands

**Keywords:** Congenital heart disease, Allograft heart valve, Valve replacement, Preservation, Tissue biobanking

## Abstract

Valvular heart disease affects 30% of the new-borns with congenital heart disease. Valve replacement of semilunar valves by mechanical, bioprosthetic or donor allograft valves is the main treatment approach. However, none of the replacements provides a viable valve that can grow and/or adapt with the growth of the child leading to re-operation throughout life. In this study, we review the impact of donor valve preservation on moving towards a more viable valve alternative for valve replacements in children or young adults.

## Background

To treat valvular heart disease, around 50,000 semilunar valve replacements are performed each year in both adult and pediatric patients in Europe (Mylotte et al. 2013), (Butchart et al. 2005). Anomalies of the aortic or pulmonary valve that require valvular replacement occur in 30% of new-borns with congenital heart disease (Alkashkari et al. 2018). These anomalies can result in valve stenosis (restricted blood flow through the cuspal opening) or valve regurgitation (incomplete valve closure and blood backflow) (O’Donnell and Yutz 2020). Valve stenosis or regurgitation can lead to increased cardiac workload, ventricular dysfunction and congestive heart failure (Mrsic et al. 2018).

To prevent progression towards heart failure in patients with congenital semilunar heart valve defects, there are several options to replace the diseased valve, albeit these are mainly fit for the adult population. These include: mechanical valves, bioprosthetic valves, pulmonary autograft in combination with an allograft valve (Ross procedure) and allograft valves. For the regurgitating aortic valve, autologous pericardium tissue has been attempted and deemed successful in multiple cases for valve reconstruction in adults (Amabile, et al. 2022) and young adults(Odim et al. 2005). For the mitral valve, valve reconstruction is more often performed than valve replacement (in 75.4% of the cases in the Netherlands) (Siregar et al. 2014). In neonates and children, repair of semilunar valves using autologous tissue is the preferred option over replacement (Hammer et al. 2017). Unfortunately, surgical repair of semilunar valves is often not possible or has to be followed by valve replacement (Hawkins et al. 2007). In children, durability of the available valve substitutes is the main concern as valve size and performance are directly related to growth of the heart resulting in patient valve mismatch. Although still debated, the Ross procedure, placement of the pulmonary autograft in the aortic position and an allograft valve in the pulmonary position, is considered the preferred valve replacement in children (Etnel et al. 2016; Takkenberg et al. 2005). The pulmonary autograft is vital, the right size, has optimal hemodynamic properties and has the potential to grow in diameter with the growing heart. Furthermore, in contrast to mechanical or bioprosthetic valves, the pulmonary autograft and allograft offer good hemodynamic performance, decreased risk of endocarditis and do not require the use of anticoagulation (Lupinetti et al. 2003; Nappi et al. 2018). Additionally, a study comparing mechanical valve replacement versus human tissue allografts showed children with allograft valves have longer survival and freedom from valve-related complications compared to children after mechanical valve placement in the aortic position (Lupinetti et al. 1999). Nevertheless, early failures of allograft valves within less than 10 years have been attributed to structural degeneration, immunologic responses and loss of cellular viability and valve growth capacity (Takkenberg et al. 2002), (Junnil et al. 2021). Autograft failure due to dilation of the valve instead of growth due to the increased mechanical stress at the aortic position compared to the pulmonary position has been reported (Etnel et al. 2018). To increase the duration of storage and increase the availability of donor valves, cryopreservation protocols were developed for tissue banking of valve allografts (Jashari 2021). Cryopreservation of valves initially showed no significant structural deterioration compared to fresh valves (Burkert et al. 2021; Witten et al. 2021). However, extensive investigation of matrix composition and cell survival after implantation revealed loss of structural organization of collagen within the valve layers and cellular damage which could predispose the valves for early structural failure (Schenke-Layland et al 2007), Schenke-Layland et al. 2006). Improving the current method of valve allograft preservation in tissue banks to better maintain valve structural integrity, function, and viability would greatly benefit allograft valve durability and limit the need for re-intervention in children with congenital heart valve defects. The ultimate goal should be to preserve vitality of the allograft, i.e., including cell viability, so that it maintains the ability to grow and remodel when implanted in the heart of a child.

In this review, we discuss current methods, problems and optimizations of semilunar valve preservation to move towards a more viable donor valve alternative for transplantation in children.

## Hallmarks of heart valve preservation—What needs to be preserved?

**Fig. 1:**
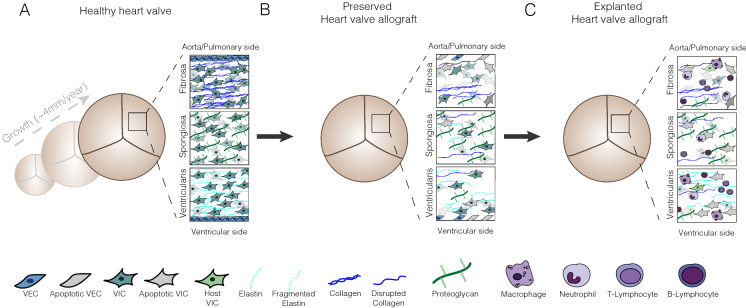
Effect of tissue processing and preservation on heart valve allograft durability. **A** Schematic representation of healthy vital heart valve with growth capacity. **B** Schematic representation of heart valve allograft after harvesting and preservation (either short-term preservation at 4 °C or long-term cryopreservation) showing loss of the structural differences of the three valve layers, elastin fragmentation, collagen disruption, and loss of VECs and VICs. **C** Schematic representation of cryopreserved heart valve allograft after transplantation showing infiltration of immune cells, a low number of host interstitial cells, and complete loss of the endothelial layer. VEC, valvular endothelial cell; VIC, valvular interstitial cell

Heart valves open and close over 3 billion times during a lifetime to maintain unidirectional blood flow from the heart to the lungs and to the rest of the body (Schoen 2011). To achieve this, semilunar heart valves are organized in three structural layers with different extracellular matrix (ECM) compositions: the collagen-rich fibrosa, proteoglycan-rich spongiosa and elastin-rich ventricularis layer (Valk et al. 2018) (Fig. 1A). The presence of valvular endothelial cells (VECs) and interstitial cells (VICs) maintains integrity of the valves and enables valve growth and remodelling during life. The guiding principles of heart valve preservation are to maintain function, shape and viability of the heart valve. To achieve this, the components of the valve (i.e. the cells, ECM and the structural organization) need to be preserved.

### The role of valvular endothelial cells

VECs line the valvular surface that is in contact with circulating blood. Their function relates to shear stress and maintaining the non-thrombogenic surface of each side of the valve that is exposed to distinct hemodynamic profiles (Schoen 2008; Ayoub et al. 2017). Additionally, communication between VECs and VICs located in the structurally distinct valvular layers mediates valvular flexibility. VECs as the mechanical sensor of the valve respond to changes in shear stress and flow and to nerve innervation (El-Hamamsy et al. 2009; Marron et al. 1996). Despite the prominent role of VECs in valve function, analysis of allograft valves showed little to no intact VECs on the surface of the valves prior to transplantation (Kamp et al. 1981), (Pfitzner et al. 2018) and of explanted failed allograft valves (Koolbergen, et al. 1998; Hawkins et al. 2003) (Fig. 1B, C). It has been proposed that the methods used to preserve and sterilize allograft valves cause loss of the valvular endothelium (Fabian, et al. 2022; Krs et al. 2004). Increasing the abundance and viability of VECs is considered an important step to increase durability of valve allografts after transplantation. However, the presence of donor VECs on the outer layers of the leaflet that are exposed to blood flow (leaflet surface) also contribute to allograft immunogenicity and immune-related graft rejection has been observed in neonates and infants (Hawkins et al. 2003; Rajani et al. 1998). Whether increasing the survival of VECs on the leaflet would be beneficial for valve performance by protecting the valve or would increase immunogenicity remains unclear. There is evidence that VECs could induce immune reactivity due the expression of immunogenic epitopes (αGal and human leukocyte antigen (HLA) proteins) (Johnson et al. 1997), (Farivar et al. 2003b), (Simon et al. 1998). However, the already observed immune reactivity to preserved valves is not related to VECs as they contain little to no remaining VECs. If immunogenicity of VECs could be prevented, improving viability and integrity of the VEC layer would be an important factor to improve allograft integration and durability.

### The role of valvular interstitial cells

Single cell analysis of valve cellular phenotypes in post-natal development till day 30 in mice indicates VEC subpopulations remain stable while VICs undergo changes in gene expression and cell function (Hulin et al. 2019). The dynamic behaviour of VICs is necessary to maintain the structural integrity of the valve during life by regulating protein synthesis and enzymatic degradation of the ECM (Ayoub et al. 2017). By secreting cytokines, growth factors, ECM components and matrix metalloproteases (MMPs) as well as their inhibitors, VICs control the structural characteristics of the three separate valve layers. While VECs are primarily localized at the leaflet surface, VICs can be found dispersed in all valve layers (Bertipaglia et al. 2003; Rabkin-Aikawa et al. 2004a). VIC characteristics change during homeostasis, development and valve disease when they adopt a smooth muscle cell, myofibroblast or osteoblast-like phenotype to enable matrix degradation and remodeling. To illustrate, VICs are responsive to vasoactive agents by increasing collagen synthesis to mediate sustained mechanical properties of the valve during variable blood pressure. Valve layer specific alterations within VICs with changing physiological pressure were found to be induced within 75 ms to mediate a resilient dynamic valve function (Huang et al. 2007). While VICs are pivotal in the functioning of healthy valves, the highly variable nature of VICs has been considered a contributing factor to structural deterioration of allograft valves as activated VICs can contribute to valve calcification (Rabkin-Aikawa et al. 2004a; Rabkin-Aikawa et al. 2004b). Changes in ECM stiffness as a result of allograft valves processing and preservation can in turn alter the VIC phenotype via the PI3/AKT elasticity sensitive pathway (Stassen et al. 2017), (Wang et al. 2013). The combination of loss of cellularity of allograft valves and a change in the phenotype of the remaining VICs could result in valve failure. Moreover, the pulmonary autograft can cause VIC activation as a result of the altered mechanical environment at the aortic position as opposed to the pulmonary position (Rabkin-Aikawa et al. 2004a). Additionally, sex- and age-related differences in gene expression by VICs have been reported to contribute to VIC plasticity (McCoy et al. 2012; Aikawa et al. 2006). Analysis of age-dependent differences in cryopreserved heart valves show more cells in younger valves and an increased collagen cross-linking and valve stiffness in older valves (Geemen, et al. 2016). In neonates, valves contain a higher VIC density and more activated VICs compared to adults (Aikawa et al. 2006). The presence and phenotype of VICs in neonatal heart valves enables valve growth, emphasizing the importance of preserving VICs and the VIC phenotype when transplanting allograft valves in young children (Rabkin-Aikawa et al. 2004a; Anstine et al. 2016).

### The role of the valvular extracellular matrix

The composition and organization of the valvular ECM regulated by the valvular cells enables valve deformation while maintaining the structurally distinct valve layers. The three layers of the valve, the fibrosa, spongiosa and ventricularis, function independently to absorb and adapt hemodynamic force and achieve complete valve closure during each cardiac cycle (Valk et al. 2018; Kodigepalli et al. 2020). Collagen, as the main stress bearing component of the valve, provides stiffness and strength while proteoglycans stabilize and elastin mediates flexibility and recoil by permitting deformation and reformation. Remodelling of the valve ECM as a consequence of altered mechanical loading (Pant et al. 2018) or post-natal valve stratification (Hulin et al. 2019) is mediated by VECs and VICs and essential to maintain valve function throughout the millions of cycles during life of valve opening and closing (Kodigepalli et al. 2020). The valvular ECM has been the base for valve tissue engineering approaches and loss of valve ECM integrity in allograft valves negatively impacts durability (Schenke-Layland et al. 2006; Fabian, et al. 2022). The loss of cellularity of allograft valves have led to the notion that allograft valves, as they are currently preserved, function as natural scaffolds to serve as a template for cellular attachment of circulating endogenous cells (Mendelson and Schoen 2006). Comparison of the pulmonary autograft and the pulmonary allograft showed loss of the three-laminar valvular structure in the pulmonary allograft alone (Rabkin-Aikawa et al. 2004b). A significantly lower collagen content was found in pulmonary allografts with progressive collagen hyalinization and loss of cellularity. A loss of elastin fibres in the ventricular layer was observed before implantation due to preservation conditions (Schenke-Layland et al. 2006). Progressive loss of the differential phenotype of the valvular layers after implantation in combination with cellular loss majorly contributes to structural valve detoriation and valve failure (Brockbank et al. 2012).

## Classic preservation methods

### Preservation at 4 °C of “fresh” valves

For heart transplantations, hearts are preserved at 4 °C in preservation or saline solution in an ice container after which the heart is transplanted within four hours of circulatory death (Copeland et al. 2020). To optimally preserve hearts multiple preservation solutions are used ranging in composition. Additives like gluconate, acetate, lidocaine, albumin, insulin, THAM, heparin, procaine, methylprednisolone, and citrate–phosphate-dextrose have been frequently used for myocardial preservation and to minimize the adverse effects of ischemia (Demmy et al. 1997). Similarly, allograft heart valves were initially preserved at 4 °C in preservation solution for a variable duration ranging from days to weeks (Takkenberg et al. 2002; Lang et al. 1994). The composition of the preservation solution to preserve allograft heart valves varies between studies (i.e. DMEM or RPMI-1640 media supplemented with foetal calf serum or HEPES (Lang et al. 1994; Gerson et al. 2009)) although most studies do not report the preservation solutions used. It is important to note that valves preserved via this method are named “fresh valves” in literature (Schenke-Layland et al. 2006; Fabian, et al. 2022; Koolbergen et al. 2002), (O’Brien et al. 1987) despite the frequently observed loss of valve viability and loss of valve functionality compared to valves directly after extraction (Burkert et al. 2008). Therefore, we believe that preservation at 4 °C for periods up to 95 days is unlikely to reflect the functionality and viability of fresh valves.

### Cryopreserved valves

The limited availability of donor organs and the short time-window between organ removal and transplantation has pushed the transplantation field to develop alternative methods that enable long-term organ preservation. By slowly decreasing temperature in combination with agents that prevent crystallization, tissues can be cryopreserved for multiple years at ~  − 135 °C using liquid nitrogen (Lisy et al. 2017). Preservation solutions for cryopreservation contain nutrient media, foetal calf serum and dimethyl sulfoxide (DMSO) or glycerol to prevent ice crystallization (Table 1). Cryopreservation aims to inhibit cellular metabolism to prevent the activation of biological and chemical processes that cause ex vivo tissue degradation and cell death (Taylor et al. 2019). After long-term storage, cryopreserved tissue can be quickly thawed and washed to remove cryoprotective agents (e.g., DMSO/glycerol) before transplantation. Using freezing containers that control the rate of freezing by lowering the temperature with 1 °C per minute accompanied by DMSO-containing nutrient media is now the main method of cryopreservation to preserve human allograft heart valves (Angell et al. 1989), (Mirabet et al. 2008).

**Table 1:** Allograft valve preservation conditions and reported performance

Preservation technique	Storage conditions (°C, duration, solution)	Valve	n	Age of recipient (mean age in years)	Implantation time	Main results	Study
4 °C and Cryo	4 °C: Donation after circulatory death, 4 °C, low-dose antibiotics Cryo: 4 °C, 24 h low-dose antibiotics prior to cryopreservation in nutrient media	Aortic	124 192	48 54	13,1 years	4 °C: Freedom from reoperation within 10 years of 84%; time of storage prior to implantation was not related to degree of valve degeneration Cryo: Freedom from reoperation within 10 years of 92%; continuing viability was seen up to 9 years after surgery	O’Brien (1987)
4 °C and Cryo	4 °C: 4 °C, antibiotic sterilized Cryo: 4 °C, cryopreservation within 21 h, 6-h low-dose antibiotic treatment	Aortic	124 (41♀) 410(164♀)	50 56	20 years	4 °C: After 14 years 50% valve incompetence; structural detoriation in 47 patients; loss cellularity Cryo: Donor cells in valve rather than repopulation with host cells; improved survival compared to storage at 4 °C	O’Brien (1991)
4 °C and Cryo	4 °C: 4 °C, 3 weeks, RPMI-1640, FCS, gentamicin, polymyxin, vancomycin, clindamycin, amphotericin Cryo: 12–24 h at 4 °C, antibiotic media + 10% DMSO for 3 weeks	Aortic	3 3	–	–	Maintained mitochondrial function; decrease cell numbers; no tissue grew in culture after preservation	Lang (1994)
4 °C and Cryo	4 °C: 4 °C, antibiotic sterilized Cryo: 4 °C, cryopreservation within 21 h, 6-h low-dose antibiotic treatment	Aortic	1022		20 years	No difference in freedom from SVD between storage at 4 °C and cryo.	O’Brien (2001)
4 °C and Cryo	4 °C: 4 °C, 28–95 days (average 32 days) Cryo: 10% DMSO (n = 22) or glycerol (n = 9)	Aortic, pulmonary	9 31	18 18	2 weeks–16 years	Implanted in RVOT; Loss of tissue architecture and cellular elements, loss collagen (elasticity), no endothelial cells; no IgG or C3 depositions or increased leukocyte adhesion molecules	Koolbergen (2002)
4 °C and Cryo	- Warm ischemia 37 °C; 12-48 h - 4 °C; saline solution; 24 h - Antibiotic treated; 24 h at 37 °C - Cryo: 10% DMSO in E199 6–38 months	Aortic, pulmonary	6 2 5 6	–	–	Initial separation of VECs; loss intercellular contact Patchy loss VECs leaflet surface; damage of lamina Total loss of VECs on leaflet surface Complete loss of VECs on leaflet surface	Burkert (2008)
4 °C and Cryo	4 °C: DMEM low glucose + glutamine + HEPES; 4 °C 3–72 h Cryo: Pre-treatment with antibiotics (fluconazole, amphotericin B, imipenem, vancomycin, and amikacin sulfate in DMEM); 10% DMSO/10% FBS; 1–6 weeks	Aortic, pulmonary	6 6	–	–	No differences in collagen and elastin structure	Gerson (2009)
Cryo	DMSO + low dose antibiotics	Aortic	34	*No available information*	~ 17 years	Complete loss of endothelium, denucleated donor fibroblasts, exposure of collagen network to bloodstream, structurally altered valves	Angell (1989)
Cryo	Warm ischemic time < 8 h; TC-199 media + 10% FCS + 5% HEPES + 10% DMSO; 30 days	Pulmonary	12	–	–	Loss of fibroblast viability (from 91 to 86%); younger donors, higher fibroblast viability	Niwaya (1995)
Cryo	*No available information*	Aortic, pulmonary	12	–	–	Normal trilaminar structure maintained; mild autolysis, nuclear pyknosis of VICs, visible VECs present	Mitchell (1998)
Cryo	*No available information*	Aortic, pulmonary	15	< 10	1–8 days 2–11 months 1–9 years	Structural detoriation; progressive loss trilaminar structure, near complete loss VECs Fragmented elastin; loss structure and leaflet thinning; loss VICs Indistinct valve layers; no VECs or VICs; sparse presence lymphocytes	Mitchell (1998)
Cryo	*No available information*	Aortic, pulmonary	18	> 10	1–8 days 2–11 months 1–9 years	1-8 days: Structural detoriation; progressive loss of the trilaminar structure, near complete loss of VECs. 2-11 months: fragmented elastin; loss structure and leaflet thinning; loss of VICs, 1-9 years: Indistinct valve layers; no VECs or VICs; sparse presence lymphocytes	Mitchell (1998)
Cryo	*No available information*	Aortic	5	< 1	< 8 months	Explanted valves contained cellular infiltrate; aneurysmal sleeve or thickened leaflets; retracted leaflet with failure to coapt; insufficiency; presence of T- and B-lymphocytes	Rajani (1998)
Cryo	*No available information*	Aortic	50 (74♀)	10,4	4 years	Allografts showed better performance than mechanical valves	Lupinetti (1999)
Cryo	*No available information*	Pulmonary	2–16	11	2 months-8 years	Allograft endocarditis or degeneration; extensive calcification allograft wall; preserved structure of ECM light microscopy; acellular	Vogt (1999)
Cryo	DMSO/Glycerol	Aortic, Pulmonary	275 (74♀)	39,7	4,8 years	In 238 patients stenosis or regurgitation is observed and reoperation within follow-up period for 34 patients due to SVD, larger diameter related to increased structural failure	Takkenberg (2002)
Cryo	Antibiotics + nutrient media	Pulmonary	20	1,7	1–12 months	Panel-reactive antibodies for HLA class 1 and class 2	Hawkins (2003)
Cryo	*No available information*	Aortic, Pulmonary	174 (18♀)	32,8	45–68 months	No influence of ABO mismatching on the allograft failure; ross RVOT reconstruction showed lower failure than other valve allografts	Jashari (2021)
Cryo	*No available information*	Aortic	210 (60♀)	40	~ 13 years	SVD in 69 patients, smaller allograft size as predictor of reoperation; leaflet tears in pressurized areas	Nappi (2018)
Cryo	*No available information*	Aortic, Pulmonary	135 (68♀)	3	~ 7,6 years	Freedom from reoperation > 80%; conduit diameter < 18 mm indicated as an important risk factor for reoperation	Junnil (2021)
Cryo	Antibiotic treatment followed by 24 h–28d storage at 4 °C before cryopreservation	Aortic, Pulmonary	57 (31♀)	–	–	Elastic fibre fragmentation in 34 cases; interlamellar MEMA in 27 cases; no laminar medial collapse, fibrosis, calcification, neovascularization, necrosis, or haemorrhage; all cases decreased cellularity and complete loss of endothelium; stronger immune response in cases with better cellularity	Fabian (2022)

*Cryo* Cryopreservation, *FCS* Foetal calf serum, *RVOT* Right ventricular outflow tract *SVD* Structural valve detoriation, *MEMA* Mucoid extracellular matrix accumulations

### Preservation type and valve durability

The impact of preservation method in valve performance and freedom from reoperation has been studied extensively. In Table 1, different preservation techniques as studied in previous studies are compared in relation to reported valve performance and cellular viability. Currently, cryopreservation below − 80 °C using DMSO-containing preservation solution is the main method for valve preservation as it was believed to increase cellular viability and improve valve performance (O’Brien et al. 1987; O’Brien et al. 1991). However, contradictory results have been published on whether cryopreservation causes superior valve durability compared to fresh valves with studies indicating improved performance of cryopreserved allograft valves (O’Brien et al. 1991; O’Brien et al. 1995) and studies observing no differences (Lang et al. 1994; Koolbergen et al. 2002; O’Brien et al. 2001) or decreased valve structural integrity (Burkert et al. 2008). Interestingly, multiple studies analysing performance of allograft valves do not report the storage conditions including storage media or storage duration which can substantially affect valve performance (Table 1).

Even though cryopreservation in DMSO-containing preservation solution is now considered the golden standard for valve allograft preservation, a study on 1022 aortic valve replacements showed no difference between structural valve detoriation in fresh and cryopreserved valves (although duration of storage is not reported) (O’Brien et al. 2001). Of note, the difference between cryopreservation and cold static storage at 4 °C might be insignificant if cryopreservation is preceded with a long period of storage at 4 °C. To illustrate, this was reported to be the case in a recent study with a period of up to 28 days of storage at 4 °C before cryopreservation (Fabian, et al. 2022). Despite the inconsistencies in literature regarding differences in cryopreserved or fresh valves, it is important to note that neither short-term cold static storage at 4 °C nor cryopreservation leads to the implantation of viable valves with growth potential. Especially in implantations that require valves with a smaller diameter, the absence of allograft growth potential due to valve non-viability remains a big predictor of early valve failure.

## Reasons of valve failure after preservation

Both valves preserved at 4 °C and cryopreserved allograft valves that were explanted due to stenosis, valvular leakage, or non-cardiac death showed limited cellularity within 1 year after implantation which is likely to have negatively impacted the valves ability to adapt, hemodynamic properties and structural integrity (Koolbergen et al. 2002). Additionally, within this first year after transplantation, the three-layered structure of the valve was lost, which was not observed in pulmonary autografts (Rabkin-Aikawa et al. 2004b). It is important to note, the only available information on allograft histology after implantation comes from failed allografts which biases the reported results. Loss of the valvular tri-laminar structure in pulmonary allografts is likely to be related to valve preservation, ex vivo processing, the surgical procedure and immune reactivity. Multiple steps of our current methods of valve preservation contribute to the loss of cellularity and ECM integrity.

### Ischemia

The loss of valve allograft cellularity has been attributed to ischemia after harvesting and the process of valve harvesting, cryopreservation and thawing (Mitchell et al. 1998). The shift from the provision of oxygenated blood to the valves in vivo to the absence of oxygenation after circulatory death and valve harvesting leads to a period of ischemia. Arresting metabolic activity through immersion in liquid nitrogen is believed to end this period of ischemia (Messier et al. 1992). A massive loss of VECs has been observed in both fresh and cryopreserved valves as a result of valve extraction, handling and loss of oxygenated blood flow (Pfitzner et al. 2018). Controversies remain on the degree of valve viability following cryopreservation and implantation. Overall cellular viability of cryopreserved donor allograft valves has been described to exceed 50% when the period between circulatory death and cryopreservation is less than 48 h (Niwaya et al. 1995; Yap and Yii 2004). Of note, this period is often longer and it remains to be seen whether the remaining viable cells can have a beneficial function after transplantation. Longer ischemia before preservation decreased valve cellularity (Lang et al. 1994; Yap and Yii 2004). Specifically, warm ischemia time (20–24 °C) has been considered a critical determinant of cellular viability of valve allografts where 37% cellular damage was observed within 2 h after isolation, increasing to 73% after 6 h (Crescenzo et al. 1992). 24 h of warm ischemia alone was found not to cause cellular ATP depletion while the combination of ischemia with cryopreservation did (Messier et al. 1992). Of note, it remains difficult to directly link cellular viability to maintained interstitial and endothelial cell phenotype and cellular function after implantation. Additionally, heart valves in complete heart transplantations showed less structural deterioration than allograft heart valves, understandably explained by decreased ischemia and the absence of cryopreservation or long term storage during heart transplantations (Mitchell et al. 1998).

### Interstitial ice formation in cryopreserved valves

During the process of cryopreservation, intercellular ice formation increases solute concentration causing cellular dehydration as water leaves the cells due to osmosis (Elliott et al. 2017). After rewarming, the cells hydrate and the DMSO leaves the cells during washing with DMSO-free solution (Pegg 2010). While commonly used agents DMSO and glycerol can efficiently protect cells from ice formation during the process of cryopreservation if the cooling rate is controlled (Brockbank et al. 2015), ice formation in the intercellular area causes damage to the tissue ECM. Both fragmentation of ECM components and loss of the laminar organization have been associated with the process of ice crystal formation during cryopreservation (Schenke-Layland et al. 2006; Shaddy et al. 1996). In 2000, Brockbank et al. observed interstitial ice formation in 75% of the cryopreserved heart valve leaflets while using anti-crystallization agents DMSO or glycerol (Pegg 2010). The formation of intercellular ice crystals was found to primarily induce damage of the valve ECM with smaller crystals in the ventricularis than in either the spongiosa or fibrosa layer (Brockbank et al. 2012; Shaddy et al. 1996). Especially ice crystal formation in the spongiosa layer was observed causing loss of valve structural integrity in the middle of the valve. Furthermore, an observed decrease in second harmonic field signals in multiphoton imaging, indicated a loss of the native structural organization of the collagen fibres in cryopreserved valves (Shaddy et al. 1996). In order to prevent tissue damage as a result of ice formation, ice-free cryopreservation methods have been developed, so called vitrification. Here, the presence of high concentrations of cryoprotectants that interact with and replace water prevent water molecules to nucleate to form ice during cooling (Shaddy and Hawkins 2002). In contrast to cryopreservation, vitrified tissue did not show damage to the ECM indicating the correlation between ice crystal formation and damage to the tissue ECM (Welters et al. 2001).

### Immunological responses

Whether immunological responses are responsible for valve allograft failure remains elusive although calcification of allografts does suggest a role of the immune system in valve failure. A previous study comparing the structural viability and integrity of cryopreserved allograft valves at different durations after implantation showed that prior to implantation the valvular three-laminar structure was still visible with only slight loss of collagen and cellular autolysis (Mitchell et al. 1998). However, even after a short period of 1–8 days of implantation, the valve showed progressive loss of the three-laminar valve structure and the number of remaining VICs and VECs. Infiltration of inflammatory cells, including neutrophils, macrophages, and T-lymphocytes was reported to be scarce within years after implantation as measured in 16 explanted aorta valves of which 5 were explanted from patients younger than 10 years of age (Mitchell et al. 1998). On the contrary, other studies report the formation of HLA type 1 and 2 antibodies within 12 months after valve transplantation (Hawkins et al. 2003). Additionally, a study measuring HLA-antibody production 3 months after valve allograft implantation at the aortic position in children reported a strong increase in circulating HLA antibodies (Shaddy et al. 1996). Furthermore, analysis of failed homograft valves in 5 children, all failed within 8 months of implantation, showed thickened valve leaflets, interstitial cell activation and foci of T- and B-lymphocytes while 7 valves explanted from adults showed structural deterioration and calcification without inflammation (Rajani et al. 1998). It remains unclear whether the immunologic response plays a role in the increased failure of allograft valves in children compared to adults (Hawkins et al. 2003; Shaddy et al. 1996), (Welters et al. 2001) (Smith et al 1995). However, a mismatch in HLA and blood group (ABO) has been associated with accelerated allograft failure in children (Baskett et al. 2003), (Yankah et al 1987). The advantage of allograft cellular viability and the disadvantage of immunogenicity require consideration when attempting to develop a better valve alternative.

## Optimizing heart valve preservation

### Preservation solutions for cryopreservation

With the described problems of allograft heart valves preservation, there might be solutions to avoid these (Table 2). The use of additional cryoprotective agents, aside from the currently used DMSO and glycerol, have been proposed to either increase post-freezing cellular viability or increase preservation of the valvular ECM. Agents that could decrease ischemic injury, cryoinjury, or prevent endothelial cell damage have been studied (Taylor et al. 2019). These include agents that actively suppress the metabolic rate (Lesnefsky et al. 2004), (Burwell et al. 2009), apoptosis inhibitors (Ha et al. 2016), (Baust et al. 2000), (Zhang et al. 2009), prevention of cryoinjury (Amir et al. 2003), and trophic factors that improve post-thawing recovery (McAnulty et al. 2002; Ostrózka-Cieślik and Dolińska 2020). To protect against ice crystal formation during the freezing and thawing process which causes cellular damage, attempts have been made to learn from protective mechanisms of freeze-tolerant animals (Tas et al. 2021). Antifreeze proteins (AFPs) that bind ice can generate a thermal hysteresis gap by lowering the freeze temperature or can inhibit ice recrystallization to prevent the maturation of small ice crystals into larger ones (Tas et al. 2021; Olijve et al. 2016). Certain polymers, such as poly (vinyl alcohol) and sugars, emulate these traits and have been exploited to lower the amount of DMSO and glycerol for cryopreservation of e.g. red blood cells (Voets 2017). AFPs have been found to enable preservation of rat heart tissue at − 1 °C without loss of viability and myocyte structure (Amir et al. 2003). Rat livers could be preserved at − 4 °C with glycerol and AFPs which decreased structural damage and increased hepatic function (bile production) compared to glycerol alone (Rubinsky et al. 1994). Preservation at even lower temperatures as used during cryopreservation using antifreeze proteins has failed so far in maintaining tissue viability (Wang et al. 1994).

**Table 2:** Problems and solutions for allograft valve preservation

Preservation technique	Problems	Phases	Potential cause of deterioration	Potential solution
4 °C	- Structural deterioration - Immunological responses - Loss of cellular viability (VICs) and abundance - Absence of VECs	4 °C	- Ischemia - Long storage before transplantation - Low temperature - Preservation solution - Absence of native natural environment	- Perfusion with oxygenated solution - As short as possible - Higher temperature with perfusion of oxygenated solution - Better medium, serum, cardioprotective agents - Bioreactor, flow, pulsatility
Cryopreservation	- Structural deterioration - Immunological responses - Loss of cellular viability (VICs) and abundance -Absence of VECs	4 °C	- Ischemia - Long storage before transplantation - Low temperature - Preservation solution - Absence of native natural environment	- Perfusion with oxygenated solution - As short as possible - Higher temperature with perfusion of oxygenated solution - Better medium, serum, cardioprotective agents - Bioreactor, flow, pulsatility
		< − 80 °C	- Crystallization due to incomplete penetration of cryoprotective agent - Crystallization due to improper cryoprotective agent	- Longer in cryoprotective agent before cryopreservation - Vitrification or addition of other agents

*VEC* Valvular endothelial cell, *VIC* Valvular interstitial cell

### Vitrification

The discovery of ice-crystal formation in cryopreserved heart valves despite the presence of cryoprotective agents such as DMSO led to the development of ice-free cryopreservation methods (Brockbank et al. 2015). Vitrification uses high concentrations of cryoprotectant solution to induce amorphous solidification rather than crystallization and subsequently restrict ice formation (Brockbank et al. 2011; Song et al. 2000). Rat heart valve vitrification using small volumes of a vitrification solution (VS) (VS55) showed no formation of ice crystals with light microscopy, maintained 80% cell viability immediately after thawing and showed decreased calcification following transplantation compared to cryopreserved valves (Brockbank et al. 2015). Rapid cooling and warming of vitrified cryopreserved valve tissue was essential to prevent ice-crystal formation (Lisy et al. 2017). Multiphoton-autofluorescence imaging showed well-maintained ECM in the vitrified cryopreserved heart valves while standard frozen cryopreservation displayed ECM alteration and freezing artefacts. Vitrified sheep heart valves showed no immune cell infiltration after being explanted while cellular viability was maintained (Brockbank et al. 2012; Lisy et al. 2017). It remains unclear whether endothelial or interstitial cells are maintained during vitrification (Lisy et al. 2017).

Vitrification in the presence of AFPs and bioinspired mimics of AFPs has been previously investigated. The use of polyvinyl alcohol (PVA) as an additive in DMSO-free vitrification-based cryopreservation showed improved viability of umbilical cord blood-derived mesenchymal stem cells (Voets 2017). Additionally, using fish-derived AFPs in vitrification-based cryopreservation of matured murine oocytes showed an improvement in cellular structure and function compared to vitrification alone (Voets 2017). Additional use of biocompatible silk fibroin could further prevent devitrification-induced recrystallization/growth of ice during the thawing process (Fan et al. 2022) and potentially improve valve viability and structural integrity. Vitrification is considered a promising approach to improve preservation of heart valves and currently various vitrification solutions are being analysed in anticipation of future clinical trials.

### Preservation solutions for maintaining fresh valves

During heart transplantations, preservation solutions are used to maintain cellular viability. Multiple solutions are used for the preservation of hearts for transplantation with a wide variety of composition. Comparison of multiple preservation solutions currently used for pediatric heart transplants (Saline, University of Wisconsin (UW) solution, Celsior, Custodiol) showed no differential effect on patient 1-year survival (Shaw et al. 2020). However, it has also been described that the wide variety of components used in preservation solutions make it hard to determine the exact effect of each component. Furthermore, in vitro culture, VICs and VECs require distinct media composition (heparin supplementation) for the maintenance of cell phenotype which might complicate the preservation of these cells in fresh valves. Of note, cells do respond differently when embedded within their own 3-dimensional ECM environment as opposed to 2-dimensional culture on plastic. Optimising the composition of preservation solutions to increase tissue viability and structural integrity could be studied to enable preservation of fresh vital valves.

### Temperature and perfusion

Regulating storage temperature could be a way to preserve viable valves. As native heart valves are preserved in the human body, they can be preserved for a lifetime at 37 °C and in the presence of oxygenated blood perfusion. Possibly, compared to cryopreservation, heart valves could be better stored at higher sub-zero temperatures (Taylor et al. 2019). However, warm ischemic storage at 20–24 °C was found to increase heart valve cellular damage (Crescenzo et al. 1992). Maintaining porcine heart valves at higher sub-zero temperatures under non-ischemic conditions has been recently attempted and shows preserved ECM integrity and cell phenotype (Konduri et al. 2005). This could be a promising approach to improve the preservation of human heart valves for transplantation. Secondly, to decrease ischemia, perfusion with oxygenated solution might be helpful. Normothermic or hypothermic perfusion has been found beneficial in the ex vivo maintenance of the heart (Fleck et al. 2021), liver (Brockmann et al. 2009), lungs (Takahashi et al. 2021) and kidneys (O’Neill et al. 2020) suggesting it could yield beneficial effects in the preservation of heart valves. Ex vivo perfusion of the explanted heart at 37 °C (normothermic perfusion) has been found to be beneficial when the heart is transplanted in children with congenital heart defects that require more complex surgeries to prevent a substantial period of cold ischemic time that could damage the organ (Fleck et al. 2021). Next to normothermic perfusion, hypothermic perfusion at 4 °C has been implemented clinically for kidney and liver transplantations where it shows higher graft success than static storage at 4 °C (O’Neill et al. 2020). Conditioning of organs outside of the human body using perfusion systems increases the time window for transplantation and minimizes organ ischemia (Brockmann et al. 2009). However, it does require the availability of organ perfusion systems and the rapid development of the necessary expertise at clinical centres. For heart valves, perfusion would mean introducing dynamic flow of oxygen and nutrient-rich media through the valves as valves do not contain vessels where blood would flow through as seen in perfusion of vascularised organs. In addition, dynamic flow through the valves might also increase the integrity of the tissue by mimicking the natural mechanical environment of the valve (Hildebrand et al. 2004). Research into the potential of ex vivo organ culture of heart valves at physiological conditions showed promising results on biological characteristics of porcine heart valves after 48 h of ex vivo culture under dynamic conditions (Konduri et al. 2005). Furthermore, bioreactor based culture of mitral valves in the presence of flow showed improved ECM maintenance compared to mitral valves under static culture conditions (Barzilla et al. 2010). Optimising flow conditions including temperature, flow speed, volume and pulsatility could simulate valve maintenance as in vivo conditions. In the development of tissue engineered heart valves (TEHVs), bioreactor based pulsatile flow systems have been used to culture and mechanically condition TEHVs (Sanders et al. 2016). The presence of flow mimics physiological conditions and stimulates the formation of collagen networks while the mechanical stress at different locations of the valve leaflet influences collagen ultrastructure (Balguid et al. 2008). Culturing heart valves under physiological flow conditions might allow for better preservation of valve ECM ultrastructure and keep the valves conditioned to maintain complete valve opening and closing and withstand shear stress. Furthermore, introducing shear stress in valve preservation would be a method to increase the preservation of VECs as it has been found to protect VEC integrity (Schoen 2008).

## Alternatives for preservation of living human valves

Next to attempts to improve the preservation of viable heart valve allografts, other approaches to improve allograft valve performance are being investigated. The immunogenicity of heart valves as a result of cells or cellular debris led to the development of valve decellularization approaches. Both xenogeneic and human decellularized valves are being studied as valve alternatives. The lower reoperation rate after transplantation of human decellularized allografts as a result of decreased antigenicity is likely to lead to fast implementation of decellularized allografts in clinical practice (Neumann et al. 2013). Currently, clinical trials in adults are showing promising results on performance of human decellularized heart valves over standard cryopreserved valves (Waqanivavalagi et al. 2020). Even though human decellularized allograft valves are increasingly being used, the issue of cryopreservation-induced damage of the ECM remains. Additionally, the process of decellularization induces valvular damage reducing the concentrations of GAGs and disruption of elastin and collagen depending on the decellularization method used (VeDepo et al. 2017). Clinical implementation of xenogeneic decellularized allograft remains far away as a recent clinical trial in pediatric patients showed rapid valve failure and early mortality within a year after transplantation by eliciting strong immune reactivity (Simon et al. 2003).

Even though the decellularized valve alternative partly overcomes the issue of reoperation due to immune activation and structural valve degeneration, it does not solve the problem of reoperation due to valve outgrowth. Repopulation of the allograft scaffold with circulating host cells was believed to potentially lead to allograft valve vitality. The presence of host fibroblasts cells in the allograft scaffold after implantation in sheep or pigs has previously been observed (Heever 2021; Dohmen et al. 2006). However, repopulation with endothelial cells has not been observed yet, (although there are some reports of in vivo endothelialisation of TEHVs (Motta et al. 2020)), and the numbers of interstitial cells in the scaffolds remain less than in native valves. To further improve the decellularized valve alternative and achieve vital valve tissue ECM remodeling, ex vivo recellularization approaches are also being investigated (Dohmen et al. 2006; Dainese et al. 2012).

## Conclusions

Current preservation approaches used in clinical practice before the transplantation of allograft heart valves is far from ideal for pediatric patients as valve viability is lost. Studies analysing the performance of allograft heart valves often do not report important parameters that influence valve viability and structural integrity such as length of preservation, ischemic period and preservation solution and cellularity when implanted. The connection between cell viability and immunogenicity requires delicate finetuning to mediate valve growth and prevent immune mediated valve deterioration.

Advances in cellularisation approaches, perfusion techniques, bioreactors and cryobiology can aid in developing a better valve alternative for children. Specifically valve growth potential dependent on valve viability is essential in preventing the need for reintervention in children.

## Data Availability

Not applicable.

## Abbreviations

VEC: Valvular endothelial cell
VIC: Valvular interstitial cell
ECM: Extracellular matrix
HLA: Human leukocyte antigen
MMP: Matrix metalloproteases
AFP: Antifreeze proteins
VS: Vitrification solution
PVA: Polyvinyl alcohol
DMSO: Dimethyl sulfoxide
TEHV: Tissue engineered heart valves
